# Safety and efficacy of the pulsed field ablation for persistent atrial fibrillation: a meta-analysis

**DOI:** 10.1186/s12872-026-05868-9

**Published:** 2026-05-12

**Authors:** Hui Li, Qin Cui, Weiping Tao, Qinghua Qiu, Jiadong Yang, Aiwu Guo

**Affiliations:** Department of Cardiology, Nantong Rici Hospital Affiliated to Yangzhou University, No. 2 Xinghu Avenue, Nantong, China

**Keywords:** Persistent atrial fibrillation, Pulsed field ablation, Thermal ablation, Safety and efficacy, Meta-analysis

## Abstract

**Background:**

Pulsed field ablation (PFA) has emerged as an innovative non-thermal approach with significant promise for treating atrial fibrillation (AF). However, comparative data between PFA and thermal ablation (TA, including cryoballoon ablation [CBA] and radiofrequency ablation [RFA]) for persistent AF (PerAF) remain limited.

**Methods:**

A systematic search was conducted in the Medline, PubMed, Embase, and Cochrane Library databases to include all relevant studies that compared PFA with TA.

**Results:**

Six trials involving 1610 patients were included in the study. Pooled analyses revealed that PFA exerted significant advantages over TA, characterized by lower recurrence of atrial tachyarrhythmias (ATAs) (risk ratio [RR]: 0.83; 95% confidence interval [CI]: 0.70 to 0.97; *P* = 0.02), especially for AF recurrence (RR: 0.79; 95% CI: 0.65 to 0.95; *P* = 0.015). Furthermore, PFA significantly reduced procedural time (standard mean difference [SMD]: −1.25; 95% CI: −1.87 to − 0.64; *P* < 0.00001) and left atrial dwell (LAD) time (SMD: −14.21; 95% CI: −27.60 to − 0.82; *P* = 0.04). No significant differences were observed between the two groups regarding fluoroscopy time and complications.

**Conclusion:**

PFA is effective and safe for PerAF treatment, featuring shorter procedure time, LAD time and lower ATAs recurrence.

**Supplementary Information:**

The online version contains supplementary material available at 10.1186/s12872-026-05868-9.

## Background

Atrial fibrillation (AF) stands as the most frequently encountered sustained cardiac arrhythmia [1]. Over the past decade, with advances in technology and the accumulation of operators’ experience, catheter ablation has become a first-line treatment for AF [2], which is currently performed mainly using thermal ablation (TA) catheters, including cryoballoon ablation (CBA) and radiofrequency ablation (RFA) [3, 4]. However, the long-term prognosis of catheter ablation remains unsatisfactory for persistent AF(PerAF) [5]. In addition, although TA effectively achieves pulmonary vein isolation (PVI), its non-specific tissue injury may affect adjacent structures, leading to complications such as phrenic nerve injury (PNI) and pulmonary vein stenosis, or the most critical, an atrio-esophageal fistula [6, 7].

A novel non-thermal technology known as pulsed field ablation (PFA) has recently emerged [8]. PFA provides multiple advantages, including tissue-selective injury, speed, and a promising long-term durability profile [9, 10]. By delivering high-intensity electric fields, PFA triggers targeted tissue necrosis while minimizing collateral damage to adjacent structures [11]. Several studies and meta analysis have shown that PFA achieves PVI with both high success rate and low complication rate [12–14]. However, in the field of PerAF, it remains unclear whether the unique advantages of PFA can translate into definitive clinical benefits. Currently, there is insufficient targeted research evidence, and findings from existing relevant studies are inconsistent. Therefore, this meta-analysis specifically focuses on PerAF to systematically evaluate the clinical efficacy and safety of PFA, with the aim of further clarifying the value of this innovative ablation strategy in this population and providing new insights and evidence-based support for the clinical management of PerAF.

## Methods

### Data sources and search strategy

A comprehensive literature search was performed across PubMed, Embase, Medline, the Cochrane Library, and Elsevier’s ScienceDirect databases. Non-English language publications were excluded from the analysis, and since our analysis only enrolled patients with PerAF, the search strategy incorporated relevant keywords and medical subject headings (MeSH) terms, including: ((persistent atrial fibrillation) OR (PerAF)) AND ((Pulsed field ablation) OR (PFA)) AND ((radiofrequency ablation) OR (RFA) OR (cryoballoon ablation) OR (CBA) OR (traditional)). The literature search was last updated in March 2026.

### Studies selection

Two reviewers (L-H and T-WP) independently performed the study screening and selection process. The inclusion criteria were as follows: (a) patients diagnosed with drug-refractory symptomatic PerAF who underwent ablation; (b) Patients undergoing their first catheter ablation procedure; (c) Comparative studies between PFA and either RFA or CBA; (d) Studies with a sample size of at least 20 participants; (e) Studies that provided comprehensive and reliable data on procedural outcomes, complications, and follow-up for both intervention groups. Excluded from the analysis were studies with unclear study designs or group allocations, as well as conference abstracts, case reports, case series, editorials, review articles, and publications in non-English languages.

### Quality assessment and data extraction

Investigators (Q-QH, Y-XD and C-Q) assessed the study quality using the Delphi consensus criteria for randomized controlled trials (RCTs), and the Newcastle-Ottawa Quality Assessment Scale (NOS) for observational research. The NOS comprises eight items, with a maximum score of nine points, utilizing a star system to evaluate study populations, group comparability, and the exposure/outcome of interest. Studies scoring ≥ 7 on the NOS were considered high quality [15]. This systematic review adhered to the Preferred Reporting Items for Systematic Reviews and Meta-Analyses (PRISMA) guidelines and Cochrane Collaboration’s recommendations. For bias risk assessment, the Robins-I tool was applied to non-randomized studies, with bias risk plots generated using the robvis software [16]. Two reviewers extracted data collaboratively, resolving discrepancies through discussion to ensure consistency [17, 18].

### Outcome definitions

#### Procedure time

Defined as the duration from the administration of local anesthesia to the removal of all catheters [19].

#### Fluoroscopy time

Refers to the total duration of fluoroscopy from the procedure initiation to its completion [19].

#### Left Atrial Dwell (LAD) time

Denotes the period during which the catheter remains within the left atrium [20].

#### Complications

 Included all-cause mortality, pericardial tamponade, persisting PNI, stroke/transient ischemic attack(TIA), esophageal lesions, and systemic thromboembolism during the subsequent follow-up [19].

#### Atrial Tachyarrhythmias (ATAs) Recurrence

Defined as the any symptomatic or asymptomatic atrial arrhythmia (include AF, atrial tachycardia (AT) and atrial flutter(AFL)) lasting > 30 s after a blanking period of 90 days [19].

### Statistical analysis

Two independent statisticians (L-H and G-AW) performed the meta-analysis by pooling summary statistics from individual trials. For dichotomous outcomes, risk ratios (RR) with 95% confidence intervals (CIs) were calculated, while continuous variables were analyzed using standard mean differences (SMD). Statistical analyses employed random-effects models, with weighting based on inverse variance methods following DerSimonian and Laird’s approach [21]. Between-study heterogeneity was evaluated using the *I²* statistic, where an *I²* >50% indicated substantial heterogeneity. A *P* < 0.05 was considered statistically significant for all tests. We performed a leave-one-out sensitivity analysis to address heterogeneity by sequentially excluding each study from the pooled analysis. Statistical analyses were performed using Review Manager version 5.4 (The Nordic Cochrane Center, The Cochrane Collaboration, 2014, Copenhagen, Denmark) [22].

## Results

### Eligible studies

**Fig. 1:**
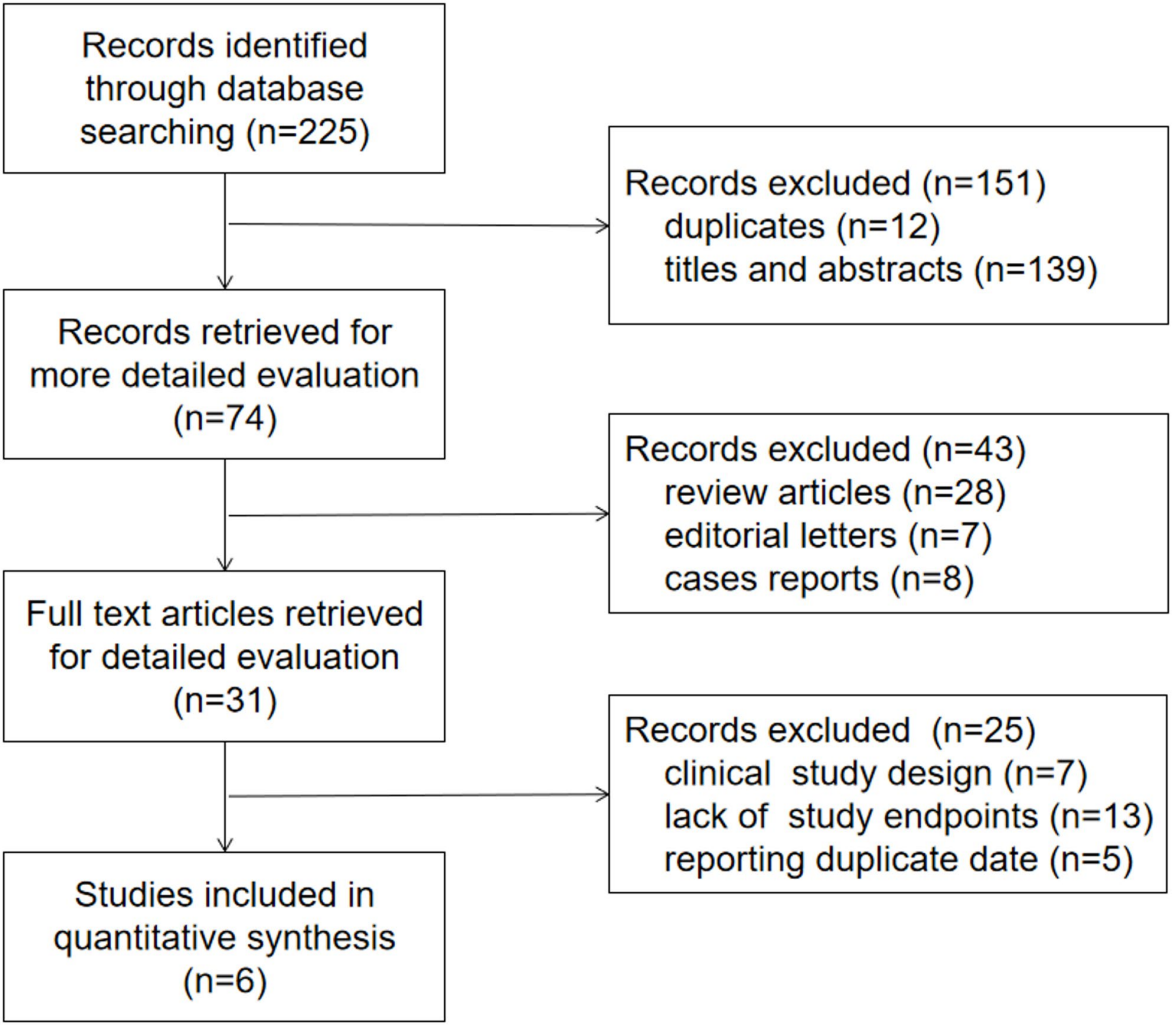
PRISMA flowchart of the screening process

The detailed literature screening process is depicted in Fig. 1. An initial search retrieved 225 potentially relevant manuscripts, of which 12 were duplicate studies and 139 were excluded after title/abstract screening. Of the 74 articles selected for full-text review, 28 systematic reviews, 7 editorials/letters, and 8 case reports/series were excluded. Further evaluation of full texts led to exclusion of 25 studies due to flawed clinical design (7 studies), missing study endpoints (13 studies), and duplicate data reporting (5 studies). Ultimately, 6 clinical trials were included in the analysis [5, 23–27]. The detailed assessment results of the NOS are presented in Table 1.

**Table 1:** The quality assessment of ten comparative studies between pulsed field ablation and traditional ablation according to the newcastle-ottawa scale criteria

Study (year)	Representativeness of the exposed cohort	Selection of the nonexposed cohort	Ascertainment of exposure	Demonstration that outcome of interest was not present at start of study	Comparability of cohorts on the basis of the design or analysis	Assessment of outcome	Was follow-up long enough for outcomes to occur	Adequacy of follow up of cohorts	NOS score
Bianchini 2024 [23]	★	★	★	★	★	★	★	★	8
Kueffer 2024 [5]	★	★	★	★	★★	★	★	★	9
Pannone 2024 [25]	★	☆	★	★	★★	★	★	★	8
Isenegger 2025 [24]	★	★	★	★	★★	★	★	★	9
Thomas 2024 [26]	★	★	★	★	★	★	★	★	8
Ahmed 2026 [27]	★	★	★	★	★★	★	★	★	9

All included comparative studies have a NOS score of 7 or higher

*Abbreviation*: *NOS *Newcastle Ottawa Scale

★ indicates 1 score, ☆ indicates 0 score

### Study characteristics

Table 2 summarizes the characteristics of the 6 included trials, involving a total of 1610 patients (765 in the PFA group and 845 in the TA group).Most trials performed patients matching between groups for age, gender, body mass index (BMI), and left atrium diameter. All included studies were assessed to have good methodological quality, supporting the validity of this meta-analysis.

**Table 2:** Baseline characteristics of included study

Study (year)	Country	Groups	Patients, (*n*)	Months since AF diagnosis, (Months)	Age, (years)	Male, (%)	BMI, (kg/m2)	Hy, (%)	DM, (%)	Stroke/TIA, (%)	CAD, (%)	LVEF, (%)	LAD, (mm)	Follow-up, (Months)	Design
Bianchini 2024 [23]	Italy	PFA	44	38.0(18.5–102)	66.0 ± 7.4	79.6	27.2[24.0–31.2.0.2]	47.5	6.8	9.1	15.9	54.3 ± 11.6	45.5 [36.5–57.5]^#^	12	Observational
		RFA	49	60.0(46–120)	63.8 ± 10.6	81.6	27.1[24.3–31.0]	52.5	0	12.5	14.3	57.1 ± 9.9	45 [38–57]^#^		
Kueffer 2024 [5]	Switzerland	PFA	214	12.0 ± 5.6	69.0[61–74]	76.6	28.1[24.9;32.3]	60.7	18.2	5.6	20.6	55.0[45.0;60.0]	45.0[38.0–53.0]^#^	12	Prospective Non-randomized
		RFA	129	9.0 ± 7.0	68.0[59–73]	69.8	28.8[25.2;32.2]	59.7	19.4	7.0	18.6	55.0[44.5;60.0]	44.0[36.8–50.8]^#^		
		CBA	190	20.0 ± 10.5	68.0[58–74]	72.6	29.0[25.1;32.7]	67.9	11.1	10.5	16.8	55.0[45.0;60.0]	40.0[31.2–50.8]^#^		
Pannone 2024 [25]	Belgium	PFA	80	10.4 ± 8.9	68.1 ± 8.6	72.5	NR	70.0	15	1.2	13.8	55.1 ± 8.2	46.2 ± 6.6	12	Retrospective
		CBA	80	11.2 ± 10.0	67.1 ± 9.0	67.5	NR	73.8	21.2	1,2	13.8	54.0 ± 7.9	45.5 ± 12.0		
Thomas 2024 [26]	USA	PFA	214	NR	69.0 [61–74]	76.6	28.1[24.9–32.3]	60.7	NR	NR	20.6	NR	NR	12	Prospective registry
		CBA	190		68.2 [58–74]	72.6	29.0[25.1–32.7]	67.9			16.8				
Isenegger 2025 [24]	Switzerland	PFA	113	NR	65.0[59–73]	78.0	27 [25–31]	59.0	10.0	NR	11.0	54[45–59]	43[39–46]	12	Observational
		CBA	107		67.0[60–71]	74.0	28 [25–31]	65,0	8.0		11.0	56[50–61]	42[39–47]		
Ahmed 2026 [27]	UK	PFA	100	16[10–30]	68[62.5–73]	72	30 ± 5.7	NR	NR	NR	NR	43.5[35.5–55.5]	48.5[42.8–55.5]	12.2 ± 2.8	Retrospective
		RFA	100	18[13–32]	68[60.5–76]	70	29.6 ± 5.1					47[40–58]	45.5[39.1–55]	12.2 ± 4.0	

Values are reported as the mean ± SD, medians (interquartile range), or *n* (%)

*Abbreviations*: *PAF *paroxysmal atrial fibrillation, *BMI *body mass index, *CAD *coronary artery disease, *DM *Diabetes mellitus, *TIA *Transient ischemic attack, *Hy *hypertension, *LAD *left atrium diameter, *NR *not recorded, *PFA *pulsed field ablation, *CBA *cryoballoon ablation, *RFA *radiofrequency ablation, *TA *Thermal ablation

^&^:vHPRF

*:LVEF < 35%

^#^:Left atrial volume index (mL/m2)

### Quality assessment

After analyzing the studies using the Robins-I tool, assessments revealed varying degrees of bias across different domains. Specifically, one study was classified as having a moderate risk of bias, with notable concerns regarding confounding bias, selection of participants bias and missing data bias [23]. The remaining studies exhibited a low risk of bias across all evaluated domains [5, 24–27]. These findings indicate that while some studies have potential flaws in confounding control and data completeness, others maintain high methodological rigor, ensuring robust research quality.

### Procedure outcomes

**Fig. 2:**
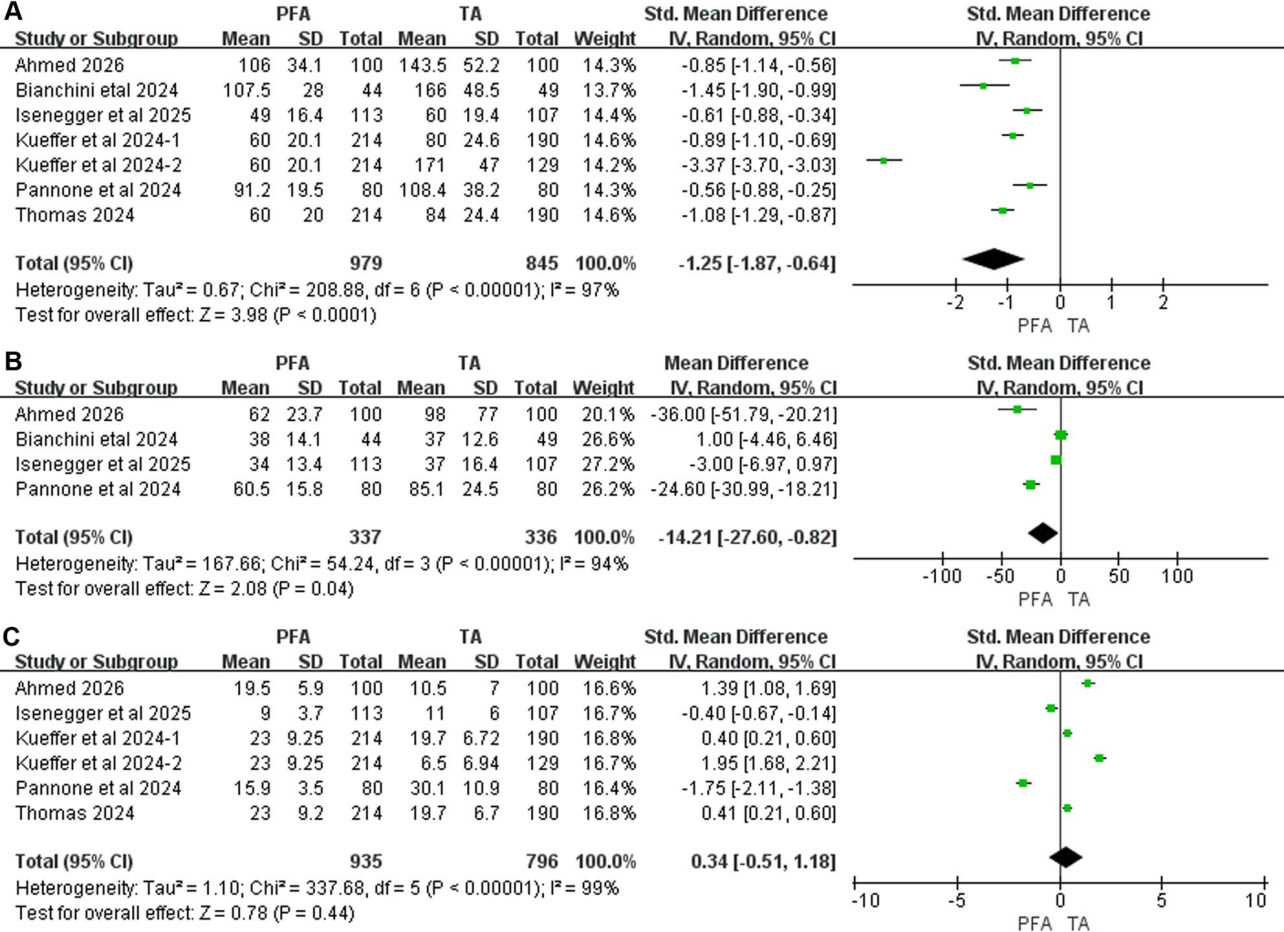
Forest plots comparing PFA and TA for procedure outcomes. **A**, procedure time; (**B**), LAD time; (**C**), fluoroscope time. CI: confidence interval; SMD: standard mean difference; PFA: pulsed field ablation; TA: thermal ablation; LAD time: left atrial dwell time; *−1*: Trials comparing between PAF and CBA; *−2*:Trials comparing between PAF and RFA

Pooled analysis demonstrated that PFA was associated with a trend of shortened procedure time (SMD: −1.25; 95% CI: −1.87 to − 0.64; *P* < 0.00001; Fig. 2A) and LAD time (SMD: −14.21; 95% CI: −27.60 to − 0.82; *P* = 0.04; Fig. 2B). No significant difference between two groups was observed in fluoroscopic time (SMD: 0.06; 95% CI: −1.26 to 1.37, *P* = 0.44; Fig. 2C). Significant heterogeneity was noted across studies for procedure time (I²= 97%), LAD time (I²= 90%), and fluoroscopy time (I²= 99%), which remained unchanged in leave-one-out sensitivity analysis. Study design (RCTs vs. observational) and ablation strategy (PVI alone vs. additional lesion sets) were further explored as potential sources of heterogeneity, but neither factor was identified as a significant moderator (Fig. S1, Fig. S2).

Subgroup analyses were performed comparing PFA with CBA and RFA separately. PFA remained associated with significantly shorter procedure time relative to both CBA and RFA (PFA vs. CBA: SMD − 0.81, 95% CI − 1.04 to − 0.57, *P* < 0.00001; Fig. 5A; PFA vs. RFA: SMD − 1.89, 95% CI − 3.52 to − 0.26, *P* = 0.02; Fig. 6A). For fluoroscopy time, no significant difference was found between PFA and CBA (SMD − 0.32, 95% CI − 1.11 to 0.47, *P* = 0.43; Fig. 5B), whereas PFA required longer fluoroscopy time compared with RFA (SMD 1.67, 95% CI 1.12 to 2.22, *P* < 0.00001; Fig. 6B). For LAD time, subgroup comparisons showed a trend toward shorter LAD time in the PFA group versus both CBA and RFA, although these differences did not reach statistical significance (Figs. 5C and 6C).

### Atrial tachyarrhythmias recurrence

**Fig. 3:**
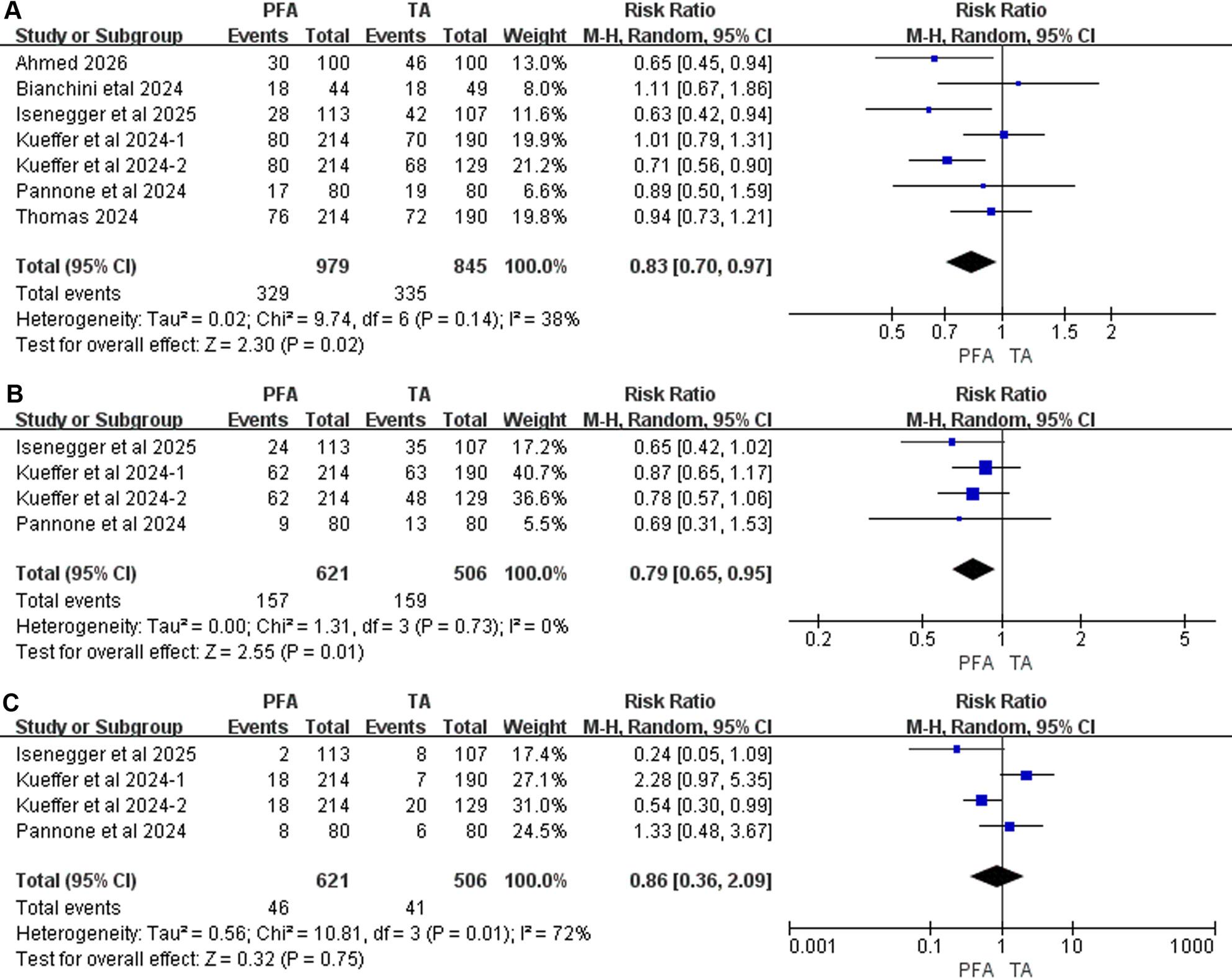
Forest plots comparing PFA and TA for recurrence. **A **,ATAs recurrence; (**B**), AF recurrence; (**C**), AT/AFL recurrence. CI: confidence interval; PFA: pulsed field ablation; CBA: cryoballoon ablation; ATAs: atrial tachyarrhythmias; AF: atrial fibrillation; AT/AFL: atrial tachycardia and atrial flutter; −1: Trials comparing between PAF and CBA

PFA was associated with a reduced risk of overall ATAs recurrence with low heterogeneity (RR: 0.83; 95% CI: 0.70 to 0.97; *P* = 0.02; Fig. 3A). A similar beneficial effect was observed for AF recurrence without significant heterogeneity (RR: 0.79; 95% CI: 0.65 to 0.95; *P* = 0.015; Fig. 3B). However, no significant difference was identified between the two groups regarding the rate of AT/AFL (RR: 0.84; 95% CI: 0.64 to 1.04; *P* = 0.10; Fig. 3C).

Subgroup analyses confirmed that PFA remained associated with a lower risk of ATAs compared with RFA (RR 0.75, 95% CI 0.59–0.96, *P* = 0.02; Fig. 6D), whereas no significant difference was observed between PFA and CBA (RR 0.89, 95% CI 0.74–1.08, *P* = 0.24; Fig. 5D). Regarding AF recurrence, PFA was associated with a reduced risk relative to CBA (RR 0.71, 95% CI 0.52–0.99, *P* = 0.04; Fig. 5E), while no significant difference was detected between PFA and RFA (RR 0.78, 95% CI 0.57–1.06, *P* = 0.11; Fig. 6E). As for the AT/AFL recurrence, no statistically significant difference was identified between PFA and CBA (RR 1.04, 95% CI 0.33–3.27, *P* = 0.94; Fig. 5F).

### Complications

**Fig. 4:**
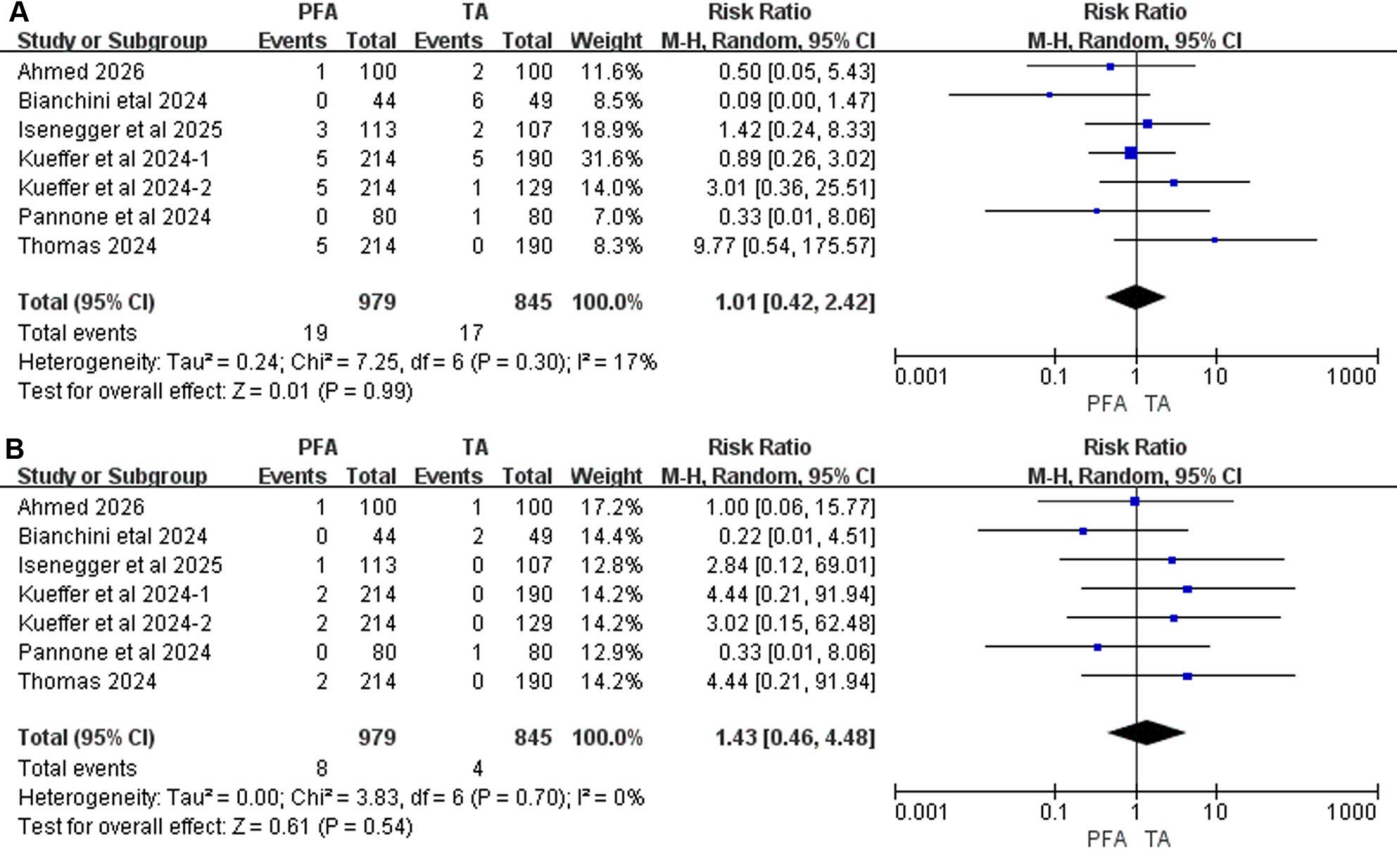
Forest plots comparing PFA and TA for complications. **A**, Total complications; (**B**), Cardiac tamponade; CI: confidence interval; PFA: pulsed field ablation; RFA: radiofrequency ablation; CBA: cryoballoon ablation; *−1*: Trials comparing between PAF and CBA; *−2*:Trials comparing between PAF and RFA

No significant difference in overall complication rate was observed between PFA and TA (RR: 1.01; 95% CI: 0.42 to 2.42, *P* = 0.99; *I²* = 17%; Fig. 4A), Consistent findings were noted for cardiac tamponade (RR: 1.43; 95% CI: 0.46 to 4.48, *P* = 0.54; *I²* = 0%; Fig. 4B). Overall, included studies exhibited low heterogeneity for complication outcomes.

**Fig. 5:**
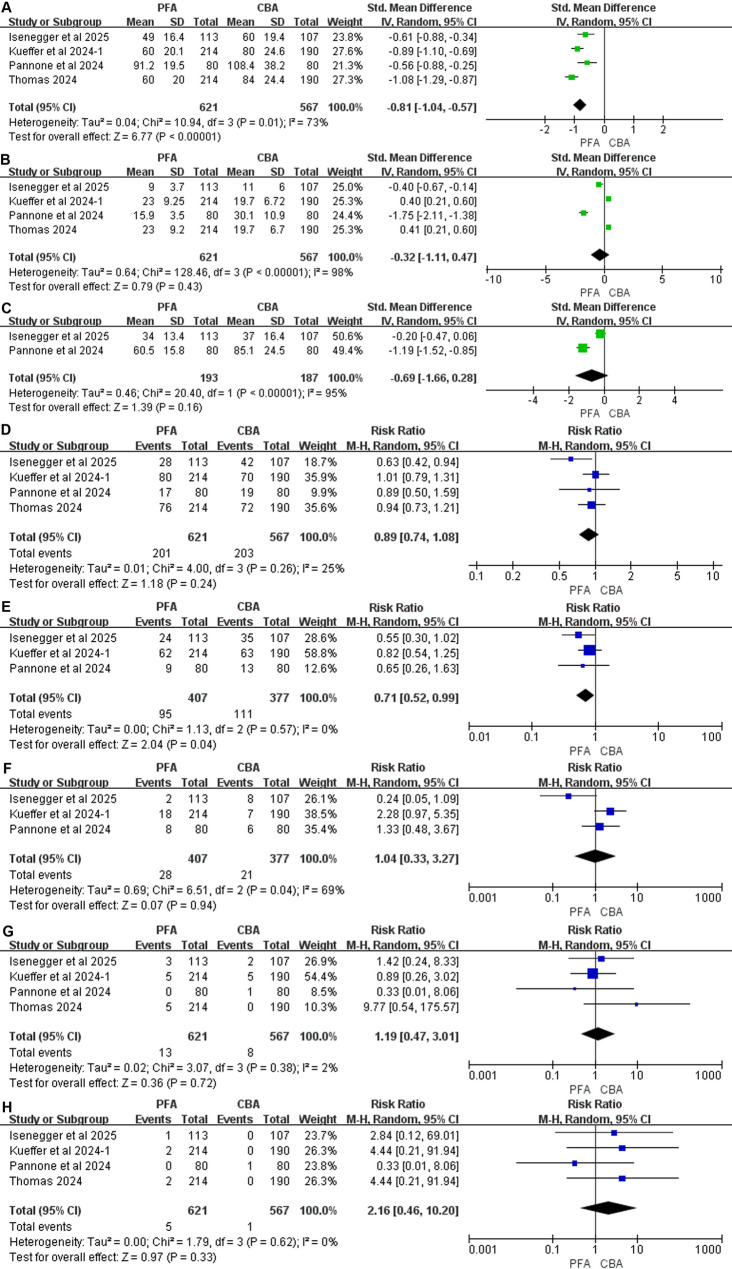
Forest plots of subgroup analyses between PFA and CBA. **A**, procedure time; (**B**), fluoroscope time; (**C**), LAD time; (**D**), ATAs recurrence; (**E**), AF recurrence; (**F**), AT/AFL recurrence; (**G**) Total complications; (**H**), Cardiac tamponade. CI: confidence interval; SMD: standard mean difference; PFA: pulsed field ablation; TA: thermal ablation; LAD time: left atrial dwell time; *−1*: Trials comparing between PAF and CBA

**Fig. 6:**
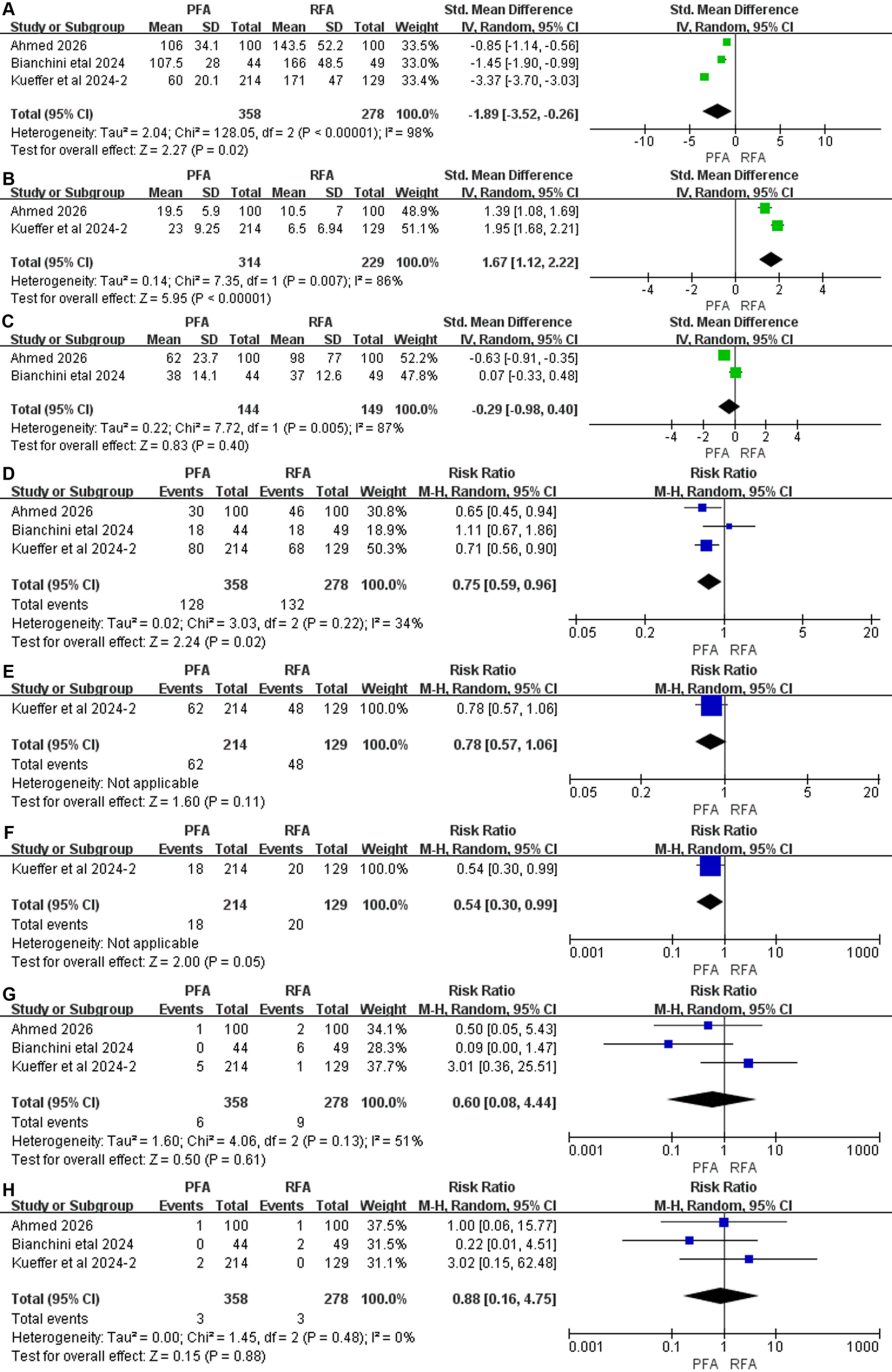
Forest plots of subgroup analyses between PFA and RFA. **A**, procedure time; (**B**), fluoroscope time; (**C**), LAD time; (**D**), ATAs recurrence; (**E**), AF recurrence; (**F**), AT/AFL recurrence; (**G**) Total complications; (**H**), Cardiac tamponade. CI: confidence interval; SMD: standard mean difference; PFA: pulsed field ablation; TA: thermal ablation; LAD time: left atrial dwell time; *−2*:Trials comparing between PAF and RFA

In subgroup analyses, PFA was not associated with an increased risk of complications compared with either CBA or RFA (PFA vs. CBA: RR: 1.19, 95% CI: 0.47 to 3.01, *P* = 0.72 Fig. 5G; PFA vs. RFA: RR: 0.60, 95% CI: 0.08 to 4.44, *P* = 0.61 Fig. 6G). Similarly, no significant difference in cardiac tamponade risk was detected for PFA versus CBA or RFA (PFA vs. CBA: RR: 2.16, 95% CI: 0.46 to 10.20, *P* = 0.33 Fig. 5H; PFA vs. RFA: RR: 0.88, 95% CI: 0.16 to 4.75, *P* = 0.61 Fig. 6H).

## Discussion

This study represents a meta-analysis and systematic review comparing the clinical outcomes using PFA versus TA in patients with PerAF. The pooled analysis yields three key findings: (a) PFA trend to reduce the recurrence of ATAs and AF; (b) PFA can significantly shorten procedure time and LAD time; (c) overall complication rates show no significant difference between the two techniques.

Our findings corroborate and extend with previous studies investing PVI in patients with PerAF in terms recurrence-free survival rates and complications for PFA. The underlying mechanism of PFA centers on non-thermal irreversible electroporation, through which ultrashort, high-frequency electrical pulses disrupt the phospholipid bilayer of cardiomyocyte membranes and form nanopores, leading to apoptosis of target atrial tissue [10, 11]. Notably, this effect has tissue selectivity: cardiomyocytes with large membrane areas and limited repair capacity are highly sensitive to electric fields, while adjacent non-cardiac structures (esophagus, phrenic nerve, etc.) are relatively resistant. Consequently, PFA achieves effective myocardial ablation while preserving extracellular matrix and vascular integrity, minimizing thermal injury, inflammation and perioperative complications [10]. Compared with thermal ablation, it attenuates inflammation and post-ablation remodeling, ensuring stable lesions and lower recurrence risks, supporting its safety and efficacy in AF pulmonary vein isolation [11]. The MANIFEST-PF study, a multicenter retrospective analysis of 1,758 patients across 24 centers, showed that PFA had no energy-related complications, shortened procedure time compared to TA [5]. and the ADVENT overall complication rates show no significant disparity between the two techniques in AF recurrence and serious adverse events [12].

Most of clinical evidence come from paroxysmal (PAF), evidence for PFA in PerAF is scarce. Kueffer et al. compared PFA with CBA and RFA in patients with PerAF. After 12-month follow-up, freedom from AF recurrence was 55% in the PFA group, 62% in the CBA group and 48% in RFA group, with improved arrhythmia-free survival observed in both the PFA and CBA groups compared to RFA [5]. Interestingly, when excluding patients with additional posterior wall isolation (PWI) in the PFA group, 1-year freedom was only 48% [5]. However, Pannone et al. compared PFA and CBA for PVI combined with PWI, with neither procedure guided by a three-dimensional mapping system. At 1-year follow-up, there was no significant difference in success rates between the two groups (76.2% vs. 78.8%; *P* = 0.63) [25]. In contrast, when comparing with RFA, Bianchini et al. found that at 1-year follow-up, the recurrence rate of ATas in the PFA group was non-inferior to that in the RFA group guided by mapping (RFA 36.7% vs. PFA 40.9%, *P* = 0.68) [23]. Therefore, for the treatment of PerAF, studies related to PFA are limited and the results are inconsistent.

### Procedure characteristics

This study’s analysis reveals that, compared with TA, PFA significantly reduces procedure time and LAD time. The efficiency of PFA can be primarily attributed to its faster completion of individual pulmonary vein ablation and higher single-shot isolation rate, underscoring the technological advancements of single-shot devices in invasive treatment of AF [28, 29]. Such rapid and efficient techniques hold promise for facilitating same-day discharge protocols following AF ablation procedures.

Non-fluoroscopic electroanatomical mapping systems are widely integrated in RFA, resulting in significantly shorter fluoroscopy time compared to CBA and PFA [12, 30–32]. In PerAF, PFA tends to require longer fluoroscopic time, primarily due to PFA requires 4 to 8 ablations for pulmonary vein, each demanding X-ray visualization for precise catheter placement [30]. However, operator experience has been shown to mitigate fluoroscopic exposure during PFA, and future integration of advanced mapping systems into PFA platforms may further curtail fluoroscopy reliance [32].

The pool analysis did reveal heterogeneity in procedure time, fluoroscopy time, and LAD time. Sensitivity analysis failed to identify a definite cause for this heterogeneity. We speculate that the heterogeneity might be attributed to differences in the proportion of PerAF across studies and variations in ablation strategies, which could contribute to the increased heterogeneity. Additionally, as multiple studies were from various global centers, differences in operators’ ablation experience may also be contributing factors. To minimize the impact of heterogeneity, we adopted a random-effects model for statistical analysis, and the trends and consistency of the results were clear.

### Efficiency

This synthesis of recent clinical evidence suggests that PFA achieves non-inferior efficacy to TA at 12-month follow-up, as validated by the MANIFEST-PF registry and PULSED AF Pivotal trial [33, 34]. The ADVENT study reported similar findings, with a 73% recurrence-free rate in the PFA group versus 71% in the TA group among patients with PAF [12]. Currently, there is a paucity of data on PerAF. Additionally, some studies have indicated that PFA may induce early afterdepolarizations and ablation edge-related reentry, with the incidence of new-onset post-ablation AT/AFL from 15% to 30% [5]. However, our meta-analysis revealed that PFA significantly reduces the recurrence of ATAs, particularly the recurrence of AF, while not increasing the risk of AT/AFL. Furthermore, recent studies indicate that, compared with conventional PFA, PFA guided by a 3D mapping system can not only further shorten procedure time, optimize target contact and localization, but also reduce the rate of arrhythmias in patients with PerAF [35, 36]. Notably, it is critical to note that operators in these trials possessed expertise in TA but limited PFA experience [31]. With evolving procedural proficiency and integration of electroanatomical mapping and intracardiac echocardiography, PFA’s therapeutic index is expected to improve.

### Safety

The comprehensive analysis suggests that complication rates in both groups remained remarkably low and statistically indistinct, aligning with prior reports [12, 37]. The diminished procedural risks are attributable to technological advancements, such as the use of contact force catheters and AI, as well as accumulated operator expertise [38, 39]. Although operators performing PFA may have less experience, the technique’s design inherently minimizes collateral tissue injury through selective targeting. In addition, compared with RFA, PFA has a shorter learning curve, with the optimization of procedures, the continuous accumulation of operators’ experience, the update of new devices, the incidence of complications is expected to be further reduced [13, 37, 40].

### Limitation

This meta-analysis has several limitations: First, publication bias could not be completely excluded, as with any literature search of databases, and inclusion of only published data contributed to bias.

Second, the number of included studies was limited to only 6 trials.

Third, most of the included studies were prospective nonrandomized, retrospective, or observational studies. Therefore, more well-designed and large-scale randomized controlled trials (RCTs) are warranted to verify the present findings. Fourth, the mean follow-up duration across included studies was approximately 12 months, indicating that longer follow-up is required to determine the long-term clinical efficacy. Fifth, significant heterogeneity was observed in procedure time, LAD time, and fluoroscopy time, the source of which could not be identified even after sensitivity and subgroup analyses. Overall, these limitations suggest the need for more comprehensive and standardized research to strengthen the conclusions drawn from this meta-analysis.

## Conclusion

This meta-analysis and systematic review demonstrated that, compared to TA, PFA trend to reduce the recurrence of ATAs and AF, shorten procedure time and LAD time, and does not increase the incidence of complications. With the continuous accumulation of procedural expertise, the safety and efficacy profiles of PFA are poised to further improve, thereby facilitating its expanded integration into clinical practice.

## Supplementary Information

Supplementary Material 1: Fig S1. Forest plots of subgroup analyses stratified by study design: comparison of procedural time and fluoroscopy time between PFA and TA., procedure time from prospective studies;, procedure time from retrospective studies;, fluoroscope time from prospective studies;, fluoroscope time from retrospective studies. CI: confidence interval; SMD: standard mean difference; PFA: pulsed field ablation; TA: traditional ablation. LAD time: left atrial dwell time; -1: Trials comparing between PAF and CBA; -2:Trials comparing between PAF and RFA.

Supplementary Material 2: Fig S2. Forest plots of subgroup analyses stratified by ablation strategy (PVI alone versus additional lesion ablation): comparison of procedural time, fluoroscopy time and LAD time between PFA and TA. (A), procedure time by ablation strategy of PVI alone; (B), procedure time by ablation strategy of PVI + additional lesion ablation; (C), fluoroscope time by ablation strategy of PVI alone; (D), fluoroscope time by ablation strategy of PVI + additional lesion ablation; (E), LAD time by ablation strategy of PVI alone; (F), LAD time by ablation strategy of PVI + additional lesion ablation. CI: confidence interval; SMD: standard mean difference; PFA: pulsed field ablation; TA: traditional ablation. LAD time: left atrial dwell time; -1: Trials comparing between PAF and CBA; -2:Trials comparing between PAF and RFA.

## Data Availability

The data that support the findings of this study are available from the corresponding author upon reasonable request.
