# Supplementary figures and images for: Safety and efficacy of the pulsed field ablation for persistent atrial fibrillation: a meta-analysis
Source: BMC Cardiovasc Disord. 2026 May 12;26:570. doi: 10.1186/s12872-026-05868-9 (PMC13340220; doi:10.1186/s12872-026-05868-9)

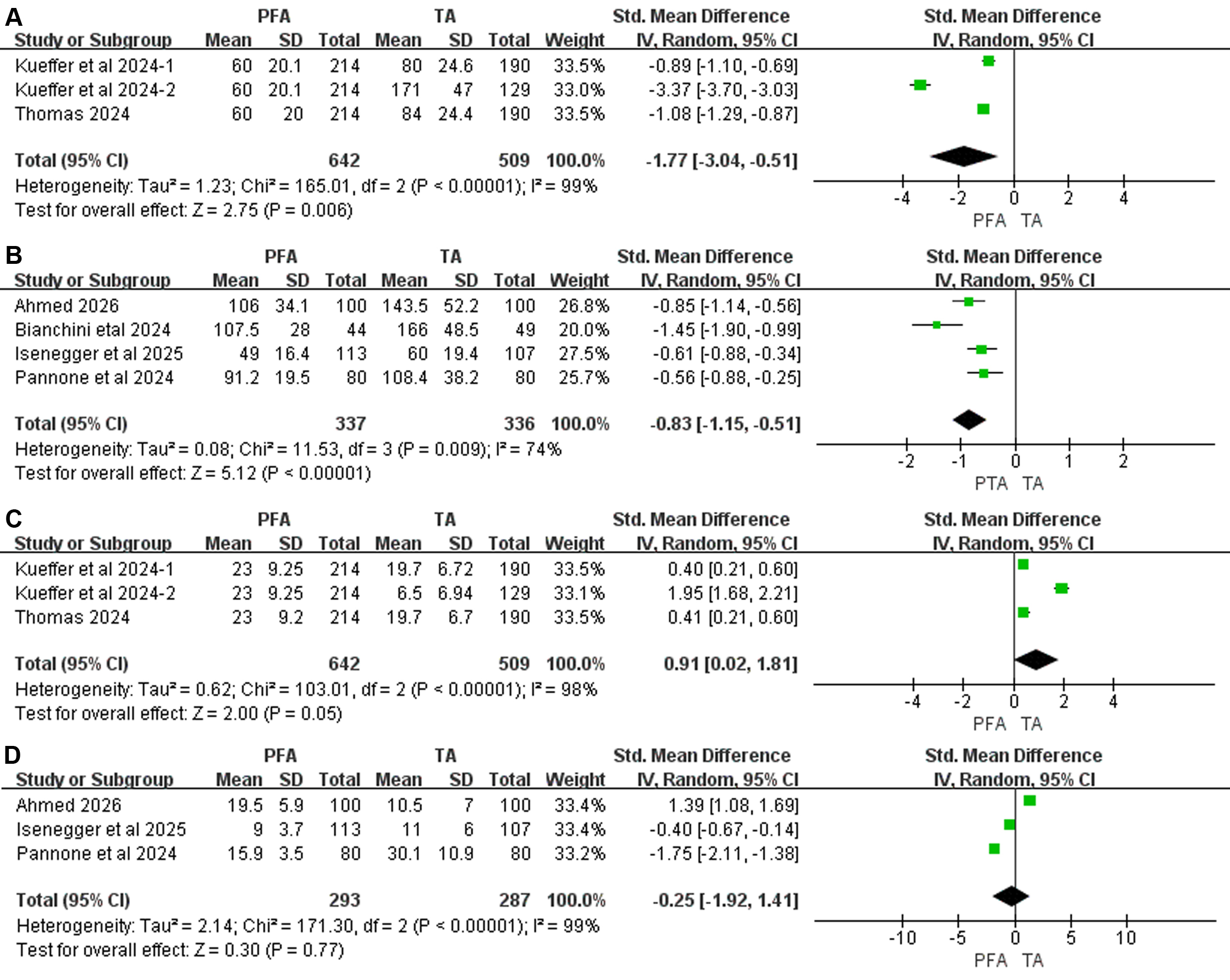

Supplement: Supplementary file 1 — Supplementary Material 1: Fig S1. Forest plots of subgroup analyses stratified by study design: comparison of procedural time and fluoroscopy time between PFA and TA., procedure time from prospective studies;, procedure time from retrospective studies;, fluoroscope time from prospective studies;, fluoroscope time from retrospective studies. CI: confidence interval; SMD: standard mean difference; PFA: pulsed field ablation; TA: traditional ablation. LAD time: left atrial dwell time; -1: Trials comparing between PAF and CBA; -2:Trials comparing between PAF and RFA. [file 12872_2026_5868_MOESM1_ESM.jpg]

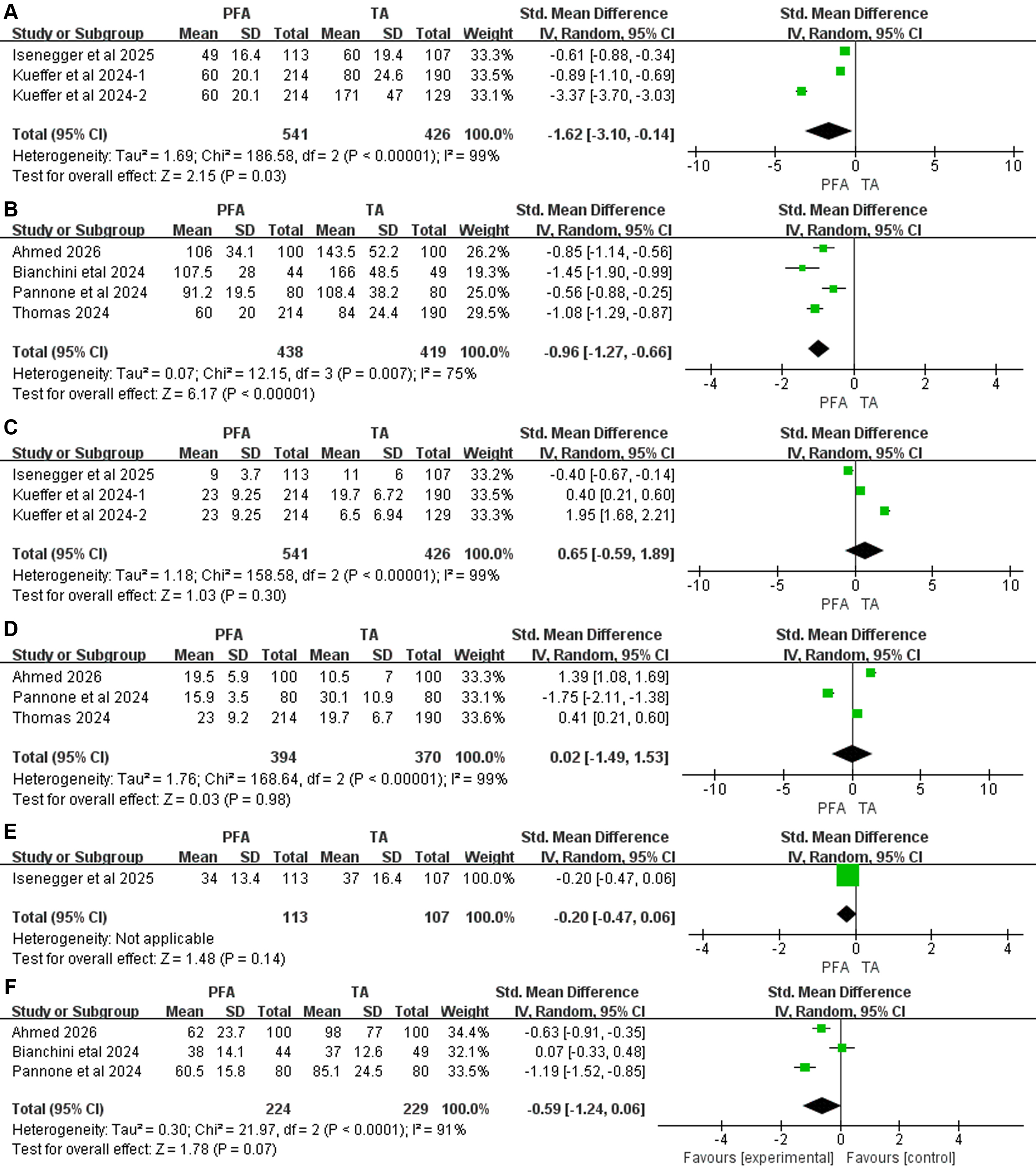

Supplement: Supplementary file 2 — Supplementary Material 2: Fig S2. Forest plots of subgroup analyses stratified by ablation strategy (PVI alone versus additional lesion ablation): comparison of procedural time, fluoroscopy time and LAD time between PFA and TA. (A), procedure time by ablation strategy of PVI alone; (B), procedure time by ablation strategy of PVI + additional lesion ablation; (C), fluoroscope time by ablation strategy of PVI alone; (D), fluoroscope time by ablation strategy of PVI + additional lesion ablation; (E), LAD time by ablation strategy of PVI alone; (F), LAD time by ablation strategy of PVI + additional lesion ablation. CI: confidence interval; SMD: standard mean difference; PFA: pulsed field ablation; TA: traditional ablation. LAD time: left atrial dwell time; -1: Trials comparing between PAF and CBA; -2:Trials comparing between PAF and RFA. [file 12872_2026_5868_MOESM2_ESM.jpg]
